# Biomimetic Alveolus-on-a-Chip for SARS-CoV-2 Infection Recapitulation

**DOI:** 10.34133/2022/9819154

**Published:** 2022-02-04

**Authors:** Ting Cao, Changmin Shao, Xiaoyu Yu, Ruipei Xie, Chen Yang, Yulong Sun, Shaohua Yang, Wangjian He, Ye Xu, Qihui Fan, Fangfu Ye

**Affiliations:** ^1^Oujiang Laboratory (Zhejiang Lab for Regenerative Medicine, Vision and Brain Health), Wenzhou, Zhejiang 325001, China; ^2^Beijing National Laboratory for Condensed Matter Physics, Institute of Physics, Chinese Academy of Sciences, Beijing 100190, China; ^3^Wenzhou Institute, University of Chinese Academy of Sciences, Wenzhou, Zhejiang 325001, China; ^4^School of Mechanical Engineering & Automation, Beihang University, Beijing 100191, China

## Abstract

SARS-CoV-2 has caused a severe pneumonia pandemic worldwide with high morbidity and mortality. How to develop a preclinical model for recapitulating SARS-CoV-2 pathogenesis is still urgent and essential for the control of the pandemic. Here, we have established a 3D biomimetic alveolus-on-a-chip with mechanical strain and extracellular matrix taken into consideration. We have validated that the alveolus-on-a-chip is capable of recapitulating key physiological characteristics of human alveolar units, which lays a fundamental basis for viral infection studies at the organ level. Using virus-analogous chemicals and pseudovirus, we have explored virus pathogenesis and blocking ability of antibodies during viral infection. This work provides a favorable platform for SARS-CoV-2-related researches and has a great potential for physiology and pathophysiology studies of the human lung at the organ level *in vitro.*

## 1. Introduction

Coronavirus disease 2019 (COVID-19) is a severe pneumonia pandemic caused by a beta coronavirus, severe acute respiratory syndrome-coronavirus-2 (SARS-CoV-2) [1]. It emerged in late 2019, and then, outbreak all over the world comes about quickly [2]. Researchers have found that SARS-CoV-2 can use its structural glycosylated Spike (S) protein for entry into host cells through binding to angiotensin-converting enzyme 2 (ACE2) receptors [3]. Its invasion brings about lymphopenia and cytokine storm in the human body with disease severity, which ultimately results in acute respiratory distress syndrome, severe pneumonia, vascular damage, gastrointestinal injury, and neurological disease [4]. However, lack of appropriate preclinical test models for SARS-CoV-2 pathogenesis accelerates the spread of the virus, which also gives rise to ceaseless invalidation of drug treatment and vaccine design, especially with the fast emergence of multiple SARS-CoV-2 variants [5]. Thus, development of a biomimetic experimental platform for recapitulating SARS-CoV-2 pathogenesis is urgent and essential for the control of the pandemic.

Conventional two-dimensional (2D) cell culture exhibits preliminary functionalities of cells but fails to reproduce microenvironment and important intracellular signaling pathways of cells [6], which might produce conflictive results for COVID-19 treatment [7, 8]. Meanwhile, animal trials face the prevailing problems of long experimental cycle and bioethical considerations and may also show different pathophysiology and clinical manifestations towards SARS-CoV-2 because of species divergences [9]. Mitigating the drawbacks of conventional research models, organ-on-a-chip as an emerging in vitro cell culture technology is proposed to precisely mimic microarchitecture and microenvironment of human organs in artificial microfluidic devices [10, 11]. It can recapitulate physiological or pathophysiological features at the organ level with high fidelity [12, 13] and is thus considered a promising strategy with dramatic advances for virology studies [14].

Lung-on-chips for SARS-CoV-2 infection studies was energetically launched recently based on the fact that the human lung is the primary target for respiratory viral infection [15]. For example, Si et al. used airway-on-a-chip to identify candidate antiviral therapeutics and prophylactics for SARS-CoV-2 [16]. Zhang et al. used alveolus-on-a-chip to explore lung injury and immune responses to SARS-CoV-2 [17]. All these lung chips are technically derived from an alveolus-on-a-chip configuration with an epithelial cell compartment, a flexible PDMS membrane, and an endothelial cell compartment initially established by Huh et al. in 2010 [18], which has biomimetic alveolar microarchitecture and microenvironment with high precision. However, the chip therein does not take into account the extracellular matrix (ECM), which plays an important role in cell adhesion, growth, proliferation, and differentiation [19, 20]. In a recent study by Zhang et al., ECM was introduced for the construction of lung-on-chips, with microchips flanking three horizontally parallel channels into lung-on-chips [21], but what is neglected in this model was the influence of air on epithelial cells, which had been proven to affect inflammatory responses of epithelial cells [18]. Therefore, the construction of a biomimetic lung-on-a-chip with consideration of both ECM and air- or fluid-induced mechanical stimulus and corresponding studies of its capabilities for SARS-CoV-2 pathogenesis are still significant and need to be investigated in detail.

Pulmonary alveoli, structural and functional units of the lung, are highly relevant to the exchange between external substances, such as SARS-CoV-2, and the human body. Here, by combining human alveolar epithelial cells, human vascular endothelial cells, collagen I, and a microfluidic chip, we developed a biomimetic alveolus-on-a-chip susceptible for SARS-CoV-2 infection recapitulation *in vitro*. This alveolus-on-a-chip consists of a human alveolar compartment, a human vascular compartment, and a sandwiched extracellular matrix compartment. Human epithelial cells and endothelial cells can be cocultured in the microchip and have reciprocal biochemical communication via porous collagen gel. Through introducing cyclic air vibration into the epithelial cell compartment to stimulate the breathing process of the lung and flowing cell medium into the endothelial cell compartment to stimulate blood flow, the alveolus-on-a-chip can recapitulate alveolar and vascular physiology and pathophysiology functioning as an innovative model for fundamental viral infection studies. Based on this alveolus-on-a-chip, we studied potential influences of mechanical forces in the microchip. We also explore inflammatory responses of the chip to poly(I:C) and the entry process of SARS-CoV-2 pseudovirus to recapitulate SARS-CoV-2 pathogenesis. This human biomimetic alveolus-on-a-chip proves to be a potential platform for recapitulating alveolar physiology and pathophysiology and can serve as a powerful tool for robust investigation of viral infection to the human lung.

## 2. Results

### 2.1. Design of the 3D Alveolus-on-a-Chip

**Figure 1:**
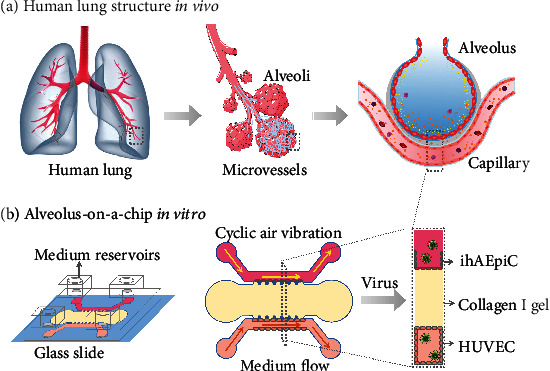
The 3D alveolus-on-a-chip platform. (a) Schematics of the alveolus and alveolar-capillary barrier *in vivo*. The alveolar unit has a key feature of epithelial cell/extracellular matrix/endothelial cell structure. (b) Design and structure of the microfluidic chip. It contains an epithelial cell (ihAEpiC) culture channel (cyclic air mechanical strain can be induced), endothelial cell (HUVEC) culture channel (continuous medium flow can be induced), and central collagen I gel channel.

As the structural and functional core unit of the lung, the human alveolus is enrobed by large capillary vessel networks. This complex architecture can be further fractured into many homologous tiny alveolar microarchitectures, which contain epithelial cells, extracellular matrix, and endothelial cells (Figure 1(a)). To recapitulate this feature, an artificial microfluidic device with three channels is used to construct a 3D alveolus-on-a-chip (Figure 1(b)) [22]. This microfluidic chip consists of a collagen gel channel in the center and two shouldered cell culture channels [23]. Medium reservoirs at the inlet and outlet ports are equipped to provide enough cell medium for cell culture. Channels are separated by trapezoid micropillars (Figure 1(b)). The micropillars are located at the border sides of the collagen gel channel so as to efficiently stabilize collagen gel structure [24].

Both ihAEpiC and HUVEC can be normally cultured in the corresponding microchambers. Biochemical molecules can permeate from one side of the microchamber to the other side spontaneously through the porous collagen gel, which is embedded in the central microchamber to guarantee fundamental biochemical compound communications between ihAEpiC and HUVEC. Specifically, both ihAEpiC and HUVEC can attach and be cultured on the collagen gel. This 3D structure gives an organ-level communication of cell-cell and cell-matrix. Besides these physiological aspects, a biomimetic alveolar microenvironment can be achieved by the addition of physical forces (Figure 1(b)). Cyclic air mechanical strain is introduced into the epithelial cell channel to simulate the breathing motion, and continuous medium flow is induced into the endothelial cell channel to simulate blood shear stress. The integration of all these factors maintains a functional microarchitecture and microenvironment of the established alveolus-on-a-chip, which can serve as a biomimetic platform for viral infection investigation.

### 2.2. Fabrication and Characterization of the Microchip

**Figure 2:**
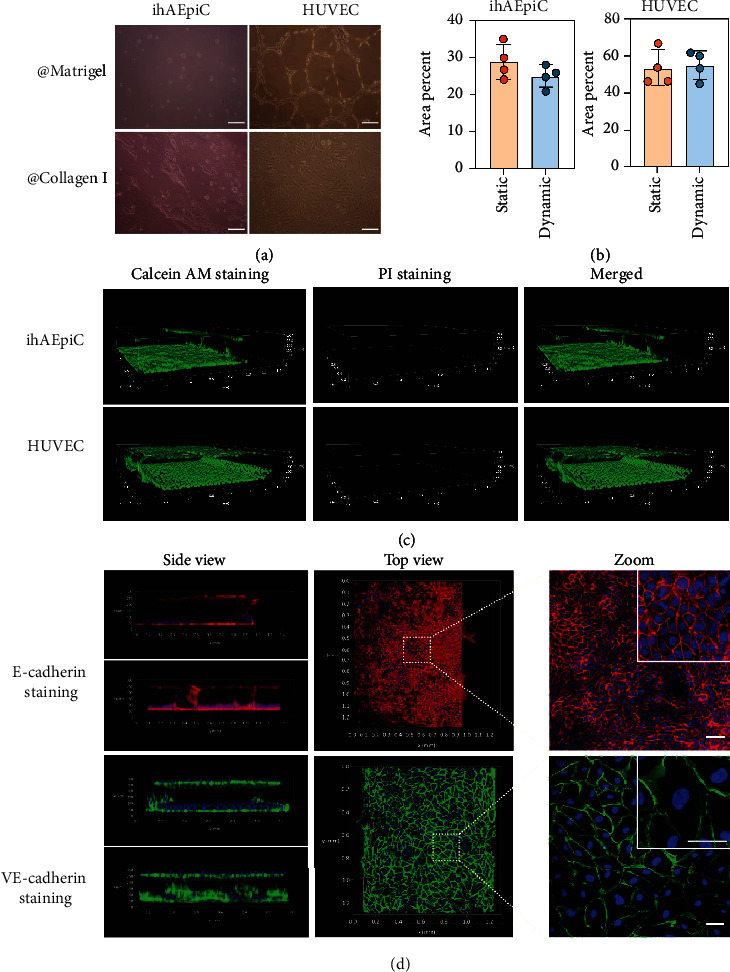
(a) HUVEC and ihAEpiC were seeded onto Matrigel and collagen I gel and normally cultured for 1 d. Scale bar is 200 *μ*m. (b) The average cell adhesion area of ihAEpiC and HUVEC on the side of collagen gel in the microchip. Error bars mean standard deviation, and each dot means one independent experiment. For the static cell culture mode, chips were normally cultured in a CO_2_ incubator and cell medium was refreshed manually every 12 h. For the dynamic cell culture mode, chips were cultured at the same experimental condition but with a flow injection pump and cell medium was continuously refreshed with an injection pump at a flow rate of 20 *μ*L/h, as shown in Figure S4 (Supporting Information). (c) The live and dead cell staining. Green fluorescence means live cells stained with calcein AM. Red fluorescence means dead cells stained with PI. (d) Result of E-cadherin and VE-cadherin immunofluorescent stain. The enlarged images show the observations under a confocal microscope with a water immersion objective. Scale bar is 50 *μ*m.

The extracellular matrix was firstly explored to obtain a favorable cell growth environment. Two common natural biological hydrogels, collagen I and Matrigel, were involved to validate their biocompatibility for the cell growth and proliferation. Both ihAEpiC and HUVEC can proliferate and spread on a collagen I gel scaffold with a normal cell morphology; in contrast, the cells on Matrigel do not exhibit normal morphology, as shown in Figure 2(a). This experimental phenomenon is in accordance with the fact that collagen is a part of the alveolar basement membrane and can support epithelial and endothelial cell adhesion [25]. During the experiments, collagen I was injected into the central channel of the chip and transformed into gel status at 37°C for 30 min. It formed a stable and uniform structure with the assistance of PDMS pillars (Figure S1, Supporting Information). As a barrier between ihAEpiC and HUVEC, collagen I gel efficiently generated two relatively independent compartments. In these two compartments, ihAEpiC and HUVEC could normally grow and proliferate with their corresponding cell culture medium, respectively (Figure S1, Supporting Information). In the meanwhile, benefiting from its porous structure, collagen I gel acted as a media for biochemical macromolecules to pass through (Figure S2, Supporting Information). Thus, an alveolus-analogous microarchitecture and microenvironment were initially established.

A cell culture manner was investigated to obtain a suitable cell culture condition for the established microchip. Here, medium reservoirs were equipped to provide cell medium for cell growth, and the medium in reservoirs was refreshed per 12 h. As a control, continuous medium flow with a flow rate of 20 *μ*L/h was also perfused into the two cell compartments. Both the static cell culture mode assisted with medium reservoirs and dynamic cell culture mode assisted with flowing medium pump supported cell growth and proliferation (Videos S1 and S2). After a period of cell culture, the cell adhesion area on the side of the collagen gel was measured by using a cell staining kit (Figure S3, Supporting Information). Four independent experiments were conducted simultaneously, and the average cell adhesion area was then calculated, with the results shown in Figure 2(b). For ihAEpiC, the static cell culture mode was slightly more favorable than the dynamic one for accelerating cell attachment to collagen gel and cell proliferation. On the contrary, for HUVEC, the dynamic cell culture mode was slightly more favorable than the static one. As a whole, there existed negligible difference between these two cell culture modes. This result proves that the medium reservoirs, functioning as a cell medium provider, could provide enough cell medium for the cell growth and proliferation in the microchip. Compared with the dynamic mode of cell culture, the static mode had simpler experimental operation and lower cell contamination probability during cell culture. Taking all these factors into consideration, the static mode of cell culture was thus chosen as the cell culture method in this work.

After cell coculture for three days with the static mode, ihAEpiC and HUVEC were stained with the calcein AM and propidium iodide (PI) cell viability assay kit. In live cells, following acetoxymethyl ester hydrolysis by intracellular esterases, nonfluorescent calcein AM can be converted to green-fluorescent calcein. In dead cells, PI can permeate into cells and nonspecifically bind to cellular DNA with a highly increased red fluorescence because of the loss of barrier capability of cell plasma membranes. As illustrated above, ihAEpiC and HUVEC were separated by a collagen gel scaffold, which supported cell growth independently and reciprocal communication of cells. The staining result stated that both ihAEpiC and HUVEC in their corresponding cell compartments could normally grow and proliferate with high cell viability. They could naturally grow into confluence and form alveolus-analogous microarchitecture in the microchip, as shown in Figure 2(c).

To demonstrate the formation of the alveolar-capillary barrier, FITC-dextran solution was injected into the epithelial cell culture channel. For this microchip without cells, FITC-dextran could permeate from one chamber to the other chamber quickly through the collagen gel (Figure S2, Supporting Information). But when ihAEpiC and HUVEC were cocultured in the chip, an obviously decreased permeability rate was observed at the same experimental conditions (Figure S5, Supporting Information). To illustrate this barrier function at the molecular level, ihAEpiC and HUVEC were stained with E-cadherin and VE-cadherin immunofluorescent analysis. E-cadherin is a cell-cell adhesion protein for epithelial cells with pivotal roles in the epithelial barrier and tissue formation [21, 26]. VE-cadherin is an important component of cell-cell adherens junctions of endothelial cells, and it is critical for maintaining the vascular integrity and endothelial barrier function [21, 27]. Figure 2(d) shows that a monolayer of the alveolar epithelium and a monolayer of the microvascular endothelium were produced adhering to the inside wall of the two cell compartments. Both epithelial cells and endothelial cells expressed markable intercellular junctional proteins E-cadherin and VE-cadherin, respectively. These two intercellular junction proteins were localized in cell-cell contact regions and formed a tight cell-cell connection.

### 2.3. Mechanical Stimulation

Previous studies have proven that air-liquid interface and physiological cyclic mechanical strain can accentuate inflammatory responses of the alveolar model *in vitro* [18]. Human lung alveolar epithelial cells with air-liquid interface can secrete surfactants, a kind of lipoproteins, to reduce the surface tension of the alveoli and maintain host defense of the lung with the stimulation of air [28]. The pulmonary surfactants are released from lamellar bodies (LB), a lysosome-related organelle with bilayer membranes [29]. LB can be stained with FM1-43 dye and act as a tool for tracking surfactant release dynamics [30].

**Figure 3:**
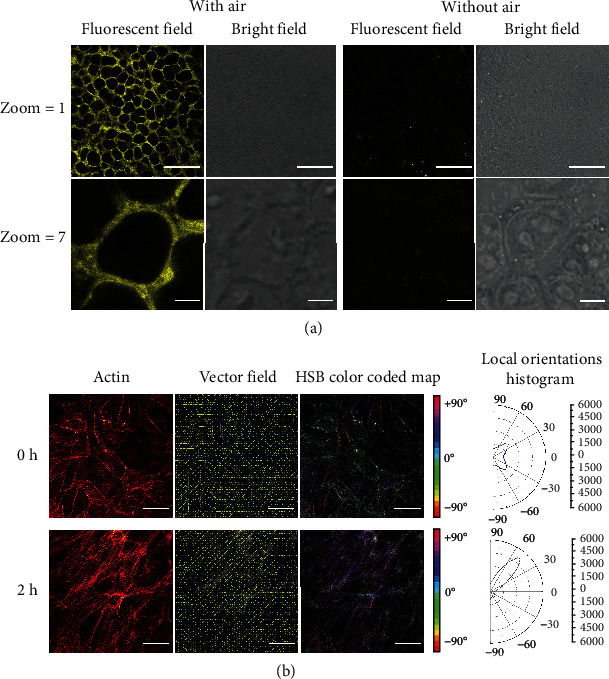
(a) Alveolar epithelial cells with/without cyclic air stimulation were stained with FM1-43 dye and viewed under a confocal microscope. Enlarged images with a large zoom magnification were shown to provide a single intact epithelial cell staining morphology. Scale bar is 50 *μ*m for zoom 1 and 5 *μ*m for zoom 7. (b) Actin was immunofluorescently stained with TRITC-phalloidin for endothelial cells stimulated with continuous medium flow at a flow rate of 60 *μ*L/h for 0 h and 2 h. Vector orientation is visualized based on the orientation of the actin image in the vector field; the orientation distribution is computed based on the structure tensor for each pixel and visualized in a color map in the HSB mode (hue is orientation, saturation is coherency, and brightness is the source image) in an HSB color-coded map. Scale bar is 50 *μ*m.

Herein, we introduced into the epithelial cells a sinusoidal cyclic air mechanical strain provided by a pressure pump to mimic breathe motion of human alveoli. The frequency and the amplitude of the cyclic air flow were set by referring to spontaneous normal breathing of an adult. The cyclic air stimulation resulted in a cyclic pressure oscillation, which could be seen from periodic vibration of the collagen gel, as shown in Video S3. Compared with epithelial cells cultured without air stimulation, alveolar epithelial cells stimulated with air stimulation produced more intracellular vesicles, which could be stained with FM1-43 dye, as shown in Figure 3(a). These stained vesicles were located in regions peripherally close to the cell nucleus (shown in Figure S6, Supporting Information) and could be kept for a long time even followed by normal medium cell culture (Figure S7, Supporting Information). According to the location, size, and property of the stained vesicles, we speculated that they might be the produced LB under stimulation by cyclic air mechanical strain. This result proved that the cyclic air stimulation did have an important influence on alveolar epithelial cells.

Human endothelial cells suffer from mechanical forces derived from pulsatile blood flow, which is essential for endothelial cell metabolism and functionalities of microvasculature. In vivo, flowing blood continuously generates fluid shear to vascular endothelial cells of the vessel lumen. In vitro, physiological fluid shear stress can induce endothelial cell alignment and elongation [31]. This endothelial cell alignment can be evaluated by cytoskeletal alignment, which plays an essential role in subcellular ECM remodeling for endothelial cells' response to mechanical stimuli [32]. We introduced a steady laminar flow of fluid medium to the endothelial compartment to observe the change of the endothelial cells. After a period of medium flow, cell elongation and alignment toward the direction of flow were observed under an inverted microscope (Figure S8, Supporting Information). Actin, a kind of cell cytoskeletal protein, was fluorescently stained so as to observe actin alignment status. Actin alignment in a randomly selected region of the microchip is given in Figure 3(b). With fluid shear stress, actin in endothelial cells showed an angle-dependent tendency. Alignment changes from an almost homogeneous distribution (with two minor peaks) to a uniform alignment whose distribution has a high peak. This uniform alignment was stable, with the presence of medium flow (Figure S9, Supporting Information). The actin alignment was also monitored in epithelial cells. However, the angle-dependent distribution of actin alignment did not exist in the stimulation of epithelial cells (Figure S10, Supporting Information). This demonstrated that fluid flow shear stress plays an indispensable role in *in vitro* investigation of endothelial cells.

### 2.4. Viral Infection Simulation

We then proceed to investigate the immune response of the established alveolus-on-a-chip treated with mechanical stimulation (Figure S11, Supporting Information). We used poly(I:C) as an immunostimulant mimicking SARS-CoV-2. Poly(I:C) is comprised of inosine poly(I) strands and cytidine poly(C) strands and has an analogous structure of double-stranded RNA (Figure S12, Supporting Information). It is a potent interferon (IFN) inducer and commonly used as an immunostimulant associated with viral infections. For example, Benam et al. used poly(I:C) to simulate the respiratory virus stimulation to examine proinflammatory response of small airway-on-a-chip [33]; Sun et al. used poly(I:C) to treat mice to explore the function of IFN-I in defending against SARS-CoV-2 and demonstrate the efficacy of poly(I:C) for COVID-19 vaccine evaluation [9].

**Figure 4:**
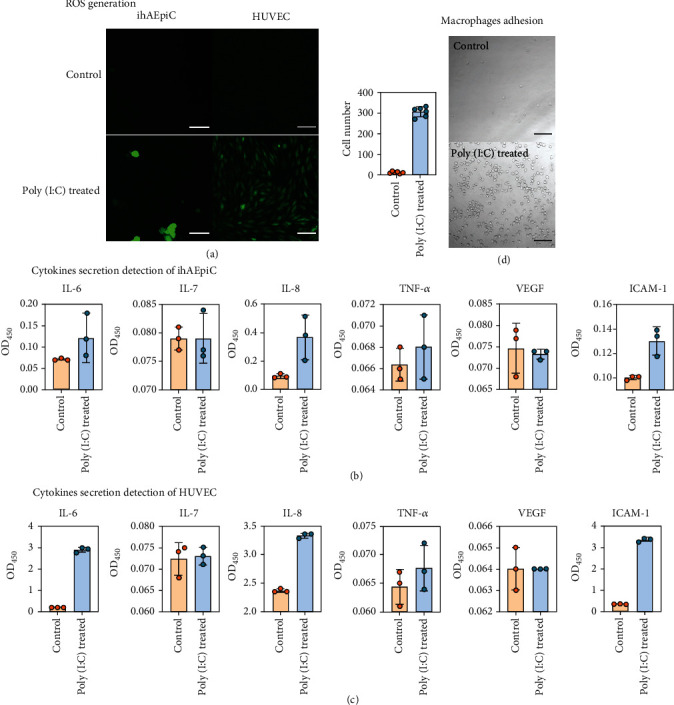
(a) ROS generation characterization before and after poly(I:C) treatment for ihAEpiC (scale bar, 50 *μ*m) and HUVEC (scale bar, 100 *μ*m). Cytokines secreted by (b) ihAEpiC and (c) HUVEC before and after poly(I:C) treatment. (d) Images of U937 cells adhering to HUVEC and number calculation of adhered U937 cells within six random areas in the microchip. Scale bar is 100 *μ*m.

We first monitored the intracellular reactive oxygen species (ROS) production of the two types of cells in the established chips. ROS is usually related to cellular oxidative stress during cell inflammatory responses [34]. Composed of oxygen radicals, peroxide, or hydroperoxide compounds in cells, ROS participates in external stimulus response in both human epithelial and endothelial cells [35, 36]. As shown in Figure 4(a), our results prove that both ihAEpiC and HUVEC give a higher ROS generation after the poly(I:C) treatment.

We then investigated the cytokine storm during viral infection. We monitored the change of IL-6, IL-7, IL-8, TNF-*α*, VEGF, and ICAM-1 before and after the poly(I:C) treatment by ELISA. IL-7, TNF-*α*, and VEGF remained almost unchanged after the treatment. In contrast, IL-8 and ICAM-1 in ihAEpiC had a significant difference after the poly(I:C) treatment, with *P* value of 0.04 and 0.01, respectively (Figure 4(b)); IL-6, IL-8, and ICAM-1 in HUVEC all significantly increased after the poly(I:C) treatment, with *P* < 0.001 (Figure 4(c)). These observations are consistent with the results of previous studies that the increase in IL-6 and IL-8 can be used as a clinical index of SARS-CoV-2 infection patients [37] and that ICAM-1 expression (related to cell adhesion) can be affected by ROS generation [36]. It is worthy to mention that in our experiments, the ihAEpiC were not as responsive as the HUVEC to the poly(I:C) treatment; we conjecture that the reason is probably related to the partial genetic changes introduced during the immortalization process of ihAEpiC [38].

As macrophages' response to viral infection is another important indicator of the immune process, we then investigated how the poly(I:C) treatment influences the behavior of U937 cells, promonocytes usually modeled as macrophages *in vitro* [39]. About 1 × 10^6^ U937 cells were added into the HUVEC compartment, to simulate free monocytes in blood (blood-circulating leukocytes). As shown in Figure 4(d) (the U937 cells on the HUVEC layer were imaged and calculated), there were only about 11 U937 cells adhering to HUVEC after a period of normal coculture of HUVEC and U937 cells; however, after the poly(I:C) treatment, about 305 U937 cells adhered to HUVEC, and the U937 cells aggregated into large masses, with an M2-like macrophage morphological characteristic, contributing to cell wound healing and cell angiogenesis [39].

### 2.5. Pseudoviral Infection

SARS-CoV-2, as a respiratory virus, has strong infectivity, high pathogenicity, and high mortality. SARS-CoV-2-related laboratory studies need to be conducted at least in BSL-3 facilities. To overcome this restriction, pseudotyped viral particles have been proposed to enable researchers without access to BSL-3 facilities to investigate basic viral biology [40]. Herein, we used a SARS-CoV-2_del19AA-GFP pseudovirus to replace native SARS-CoV-2 for the host cell entry studies [40]. This pseudovirus was facilitated by incorporating the S protein and GFP gene into lentiviral pseudovirons and at the same time removing the last 19 amino acids. It can faithfully recapitulate the key characteristic of SARS-CoV-2 infection whose S protein is used to bind with ACE2 receptors for entry into host cells.

**Figure 5:**
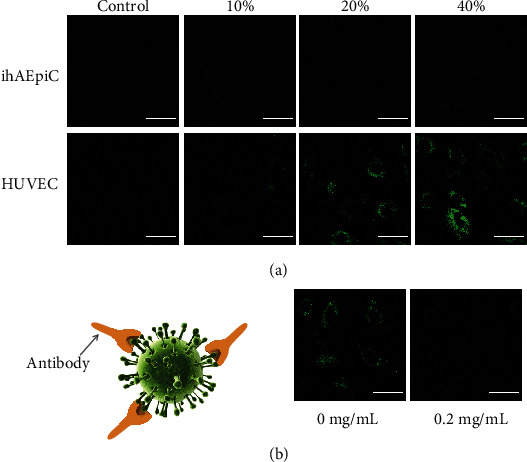
(a) Pseudoviral infection with different viral concentrations in ihAEpiC and HUVEC. (b) The influence of the SARS-CoV-2 Spike-RBD monoclonal antibody on viral infection. Scale bar is 50 *μ*m.

As shown in Figure 5(a), infection efficiency of the pseudovirus on ihAEpiC and HUVEC increased with the concentration of the virus. For ihAEpiC, no significant viral infection was observed until the virus concentration reached 40%. In contrast, for HUVEC, 10% pseudovirus could already induce infection. We note that this result is not in accordance with other works, in which epithelial cells are more susceptible to SARS-CoV-2 than endothelial cells [17]. We speculate that the reason of this difference may be related to the immortal process of human alveolar epithelial cells. We further preincubated 20% pseudovirus for 2 h with 0.2 mg/mL monoclonal antibody targeting the SARS-CoV-2 Spike receptor-binding domain (RBD) and then added the mixture into the endothelial compartment. After being cocultured with the cells for 3 d, the antibody effectively inhibited the entry of viruses into host cells (Figure 5(b)). The quantitative fluorescence intensity of the GFP for the viral infection was calculated by using ImageJ software and is shown in Figure S13.

## 3. Discussion

By introducing medium reservoirs to provide cells with nutrition supplement, collagen gel to serve as extracellular matrix support, and mechanical forces to mimic the microenvironment of cells, we established alveolus-on-a-chip that can realize coculture of epithelial and endothelial cells, reciprocal biochemical communications of cell-cell and cell-matrix, reproduction of air-liquid interface, simulation of the cyclic expansion/contraction breathing mechanism, and stimulation of mechanical flow strain. Virus-analogous poly(I:C) treatment was further conducted to investigate the inflammation and immune response. SARS-CoV-2_del19AA-GFP pseudovirus infection was also applied to mimic the viral entry process and to test the antibody inhibition ability. In summary, we established a biomimetic alveolus-on-a-chip that can recapitulate physiological and pathophysiological characteristics of human alveoli units.

Compared with a conventional 2D monolayer cell culture and animal assay, this alveolus-on-a-chip model established a 3D human alveolus microarchitecture and microenvironment. It evolves from previous alveolus-on-chips but owns the advantages of a more straightforward experimental operation and a more biomimetic alveolar structure with mechanical strain and extracellular matrix hydrogel. It reconstitutes microenvironment and functionalities of tiny alveolar units, realizes spatial cell-cell and cell-matrix interaction, and involves complex cell response to external stimulants. Although immortalized human alveolar epithelial cells were used as the model cells in our experiments because of the cell proliferation deficiency of primary alveolar cells *in vitro*, the immortalization process brought some unexpected results into our observations; this work undoubtedly provides a new thinking and platform for studies on SARS-CoV-2 infection and shows a great potential for physiology and pathophysiology studies of the human lung at the organ level.

## 4. Materials and Methods

### 4.1. Fabrication of the Microfluidic Device

The fabrication of the PDMS (Dow Corning, USA) device is followed by the standard photolithography instruction. Firstly, a silicon wafer was cleaned and deposited with an SU-8 2100 photoresist (Microchem, USA) by spin coating. Soft bake the obtained photoresist-coated silicon wafer at 65°C for 5 min and 95°C for 20 min. After cooling down to room temperature, the wafer was exposed to UV light under a printed film, which was an optimized CAD design mask (Figure S14, Supporting Information). After exposure, the wafer was postbaked at 65°C for 5 min and 95°C for 10 min. It was cooled down to room temperature again. Wash the uncross-linked SU-8 away with a developer reagent. And then a silicon master with microfluidic patterns was formed. Heat 5 *μ*L trichloro(1H,1H,2H,2H-perfluorooctyl) silane (Sigma-Aldrich, Germany) at 120°C for 5 min, and make its vapor coat the surface of the master. After cooling down to room temperature, put the master in a dish and pour bubble-free PDMS mixture (base/curing agent = 10 : 1 by weight) onto it. Cure the mixture at 60°C for 3 h. Then, peel down the casted PDMS from the silicon master and obtain a PDMS slab with corresponding microfluidic features. Punch the slab by using a 2 mm puncher manually, and bond it to a 25 × 25 mm glass slide by using a Harrick plasma cleaner. At the inlet and outlet ports, PDMS-made reservoirs were bonded with the slab by using a plasma cleaner again to form the finial microfluidic chip. A brief experimental diagram of the whole microfluidic chip fabrication has been shown in Scheme S1.

### 4.2. Cell Culture

Type II pneumonocytes have been demonstrated to have ACE2 receptors on the cell surface for SARS-CoV-2 Spike protein binding [41]. Immortalized human alveolar epithelial cells (ihAEpiC) derived from type II pneumonocytes of human lung tissue were gifted from Tongji University and normally cultured in DMEM (Gibco, USA), supplemented with 10% FBS (Gibco, USA) and 1% penicillin-streptomycin (Corning, USA). Primary human umbilical vein endothelial cells (HUVEC) were purchased from ScienCell Research Laboratories, Inc. (CA, USA). They were cultured in specific commercialized endothelial cell medium (ECM, ScienCell, USA) according to the manufacturer's instructions. Passage 3-7 HUVEC with cobblestone-like cell morphology were used during the whole experiment. All these two kinds of cells were cultured in T25 cm^2^ culture flasks and incubated under a humidified atmosphere at 37°C and 5% CO_2_.

### 4.3. Microfluidic Cell Culture

The channels of the assembled microchip were immersed in 75% ethanol for 10 min for sterilization. After that, dry the chip at 60°C for 3 h. 50 *μ*g/mL fibronectin solution (ScienCell, USA) in DPBS was freshly prepared and injected into the chip channels for coating at 37°C for 30 min. Then, the fibronectin solution was aspirated. Wash the fibronectin-coated chip with sterile H_2_O three times. Dry the chip at 40°C overnight for further usage.

DMEM and ECM were incubated at 37°C in a water bath at least for 1 h to remove bubbles and air in the medium solution. 6 mg/mL collagen I (Corning, USA) gel was prepared according to the manufacturer's instructions and injected into the collagen channel of the chip directly. Check the shape of the collagen under an inverted microscope (Nikon ECLIPSE TS100, Japan; it was equipped with a Canon camera) to make sure that it fulfilled the central collagen channel evenly. Solidify the collagen to form a collagen gel at 37°C for 30 min.

ihAEpiC were harvested and resuspended in the preheated DMEM at a density of 5 × 10^4^ cells/mL. HUVEC were harvested and resuspended in the preheated ECM at a density of 1 × 10^6^ cells/mL. Transfer the ihAEpiC and HUVEC into their corresponding cell channels with a 20 *μ*L pipette. Add DMEM and ECM into inlet and outlet reservoirs of ihAEpiC and HUVEC cell channels, respectively. Incubate the cell-seeded chip at 37°C for 1 h to promote the adherence of cells, and then, replace it with fresh medium to culture cells for another 2 h. The same procedure as the above description was repeatedly conducted for the remaining two sides of the epithelial and three sides of endothelial compartments. After that, culture the cells in the microchip at 37°C for three days, during which change the medium with fresh medium per 12 h. For dynamic flow culture condition, a LSP10-1B syringe pump with 10 channels, together with 1 mL syringe-connected PTFE tubing (1/16^″^ OD and 1/32^″^ ID) by 20 G screw syringe needles, was used to perfuse DMEM and ECM at a flow rate of 20 *μ*L/h for 48 h. Photos of the chip are shown in Figure S15.

### 4.4. Cell Viability Assay

A Viability/Cytotoxicity Assay Kit for animal live and dead cells (calcein AM, PI) was bought from US EVERBRIGHT (Suzhou, China) and used to obtain cell viability result. Firstly, aspirate the cell medium from the chip and wash the cells with fresh cell medium three times. Then, fresh medium containing cell stain dye (0.5 *μ*L 4 mM calcein AM and 3 *μ*L 1.5 mM PI in 1 mL DMEM/ECM) was injected into the cell channels in the chip and incubated at 37°C for 20 min. After that, wash the channels again with fresh medium three times and image the pictures under a confocal microscope (Leica TCS SP8, Germany).

### 4.5. Permeability Assay

10 *μ*M 4 kDa FITC-dextran solution (in DMEM) was delivered into the epithelial compartment of the cell-cultured microchip. Fluorescence intensity was recorded in real time at different sites for evaluating cell compactness and permeability of the alveolar-capillary barrier under an inverted microscope (Nikon ECLIPSE Ti, Japan). A blank microchip without cells was also monitored under the same experimental condition.

### 4.6. Immunofluorescence Analysis

Aspirate DMEM and ECM from the chip, and wash them with preheated fresh cell medium three times. Then, fix epithelial and endothelial cells with 4% preheated paraformaldehyde (in PBS) at room temperature for 30 min. Wash away the paraformaldehyde with PBS three times. Keep the PBS solution in the chip channels for 5 min, and repeat the wash step three times. Permeabilize cells with 0.5% (*v*/*v*) Triton X-100 (in PBS) at room temperature for 10 min. Then, wash cells three times with PBS. Incubate the permeabilized cells with 3% BSA (in PBS) for blocking nonspecific antibody binding sites at room temperature for 30 min. Following that, a primary E-cadherin antibody (mouse mAb, 1 : 50 diluted in PBS, CST) and primary VE-cadherin antibody (rabbit mAb, 1 : 200 diluted in PBS, CST) were incubated with cells at 4°C overnight in the epithelial cell compartment and endothelial cell compartment, respectively. Wash them with PBS three times again. Subsequently, goat anti-mouse Alexa Fluor 647 (1 : 100 diluted in PBS, Abcam) and goat anti-rabbit Alexa Fluor 488 (1 : 100 diluted in PBS, Abcam) secondary antibodies were injected into the corresponding cell channels and incubated with cells at room temperature for 2 h. They were washed with PBS three times. Stain the cell nucleus with Hoechst 33342 solution (1 : 500 diluted in PBS, Thermo Fisher) at room temperature for 15 min. After PBS washing, fluorescent images were acquired by using a confocal fluorescent microscope and further treated by using Fiji software.

### 4.7. Microenvironment Simulation

For ihAEpiC, a stable cyclic air flow powered by a pressure pump was provided to mimic alveolus spontaneous breathing. A baseline air pressure of 5 mbar and a hyperinflation air pressure of 20 mbar with a sinusoidal waveform were applied to the epithelial compartment at a frequency of 0.2 Hz for 10 min. At the same time, a medium mixture (DMEM/ECM = 1 : 1) was perfused into the endothelial compartment with a flow rate of 20 *μ*L/h. FM1-43 (NerveGreen C4), a lipophilic styryl molecule with an amphiphilic ability involving lipid membranes, was used to label LB for the indication of surfactant release [30]. It was purchased from US EVERBRIGHT (Suzhou, China) and stored at -20°C. 5 *μ*M FM1-43 in DMEM was injected into the epithelial compartment after air flow stimulation and incubated for 10 min. It was washed with fresh DMEM three times and imaged under the confocal fluorescent microscope.

For HUVEC, ECM was perfused into the endothelial cell compartment with a flow rate of 60 *μ*L/h for 2 h. RITC Phalloidin (Solarbio, China) was used to stain actin in cells with the same immunofluorescence analysis procedure. A structure tensor for each pixel was considered to evaluate actin orientation. An orientation vector field and distribution were then analyzed by using an ImageJ plugin, Orientation J (http://bigwww.epfl.ch/demo/orientation/).

### 4.8. Poly(I:C) Stimulation

Polyinosinic-polycytidylic acid, abbreviated as poly(I:C), was purchased from InvivoGen (USA). 20 mg/mL poly(I:C) stock solution was prepared with endotoxin-free physiological water and split into 50 *μ*L aliquots. Store them at -20°C, and avoid repeated freeze-thaw cycles. 100 *μ*g/mL LMW poly(I:C) was diluted in DMEM/ECM and incubated with cells for 12 h. Then, wash it with fresh medium for further usage or detection.

### 4.9. ROS Analysis

Intracellular reactive oxygen species (ROS) generation level was measured by using a Reactive Oxygen Species Assay Kit (Solarbio, China). Wash the microchip with fresh medium three times, and then, add 10 *μ*M DCFH-DA (diluted in DMEM/ECM). It was incubated at 37°C for 30 min. Wash the DCFH-DA solution away with fresh medium, and detect fluorescence intensity immediately using the confocal fluorescent microscope.

### 4.10. Cytokine Quantification

Cell medium was collected into tubes and stored at -20°C until further usage or detection. Released cytokines IL-6, IL-7, IL-8, TNF-*α*, ICAM-1, and VEGF in cell medium were detected using human cytokine ELISA detection kits (Neobioscience, China). When detected, 20 *μ*L cell medium was used to determine the content of human cytokines according to the manufacturer's instructions. Fluorescent intensity was measured by using a 96-well microplate reader (Thermo Fisher, USA).

### 4.11. Macrophage Adhesion

U937 human myeloid leukaemia cells were bought from ATCC and cultured in RPMI-1640 medium (Gibco, USA), containing 10% FBS (Gibco, USA) and 1% penicillin-streptomycin (Corning, USA). They were cultured in T25 cm^2^ culture flasks and incubated under a humidified atmosphere at 37°C and 5% CO_2_. Following poly(I:C) treatment on HUVEC, 1 × 10^6^ U937 cells in ECM were added into the endothelial cell channels and incubated for 6 h. A control experiment without poly(I:C) treatment was conducted at the same time. After incubation, excess U937 cells were washed away from the endothelial compartment. Image adhesion U937 cells on HUVEC under the confocal microscope, and calculate the adhered U937 cell number.

### 4.12. Pseudovirus Infection

Different concentrations of 2.22 × 10^6^ TU/mL SARS-CoV-2_del19AA-GFP pseudovirus (PackGene, China) in ECM were added into the endothelial compartment when HUVEC were cultured for 12-24 h. After 2 days of viral infection, cells were washed with fresh medium and cultured for another day. GFP expression was imaged to assess viral infection on cells under the confocal microscope.

20% pseudovirus and 0.2 mg/mL SARS-CoV-2 Spike-RBD neutralizing recombinant antibody (InvivoGen, USA) in ECM were mixed evenly and preincubated at 37°C for 2 h. As a control, only 20% pseudovirus and no antibody were also used under the same experimental condition. Inject these two kinds of solution into the endothelial compartment, and infect HUVEC with the above infection procedure. After infection, detect the GFP expression under the confocal microscope.

## Data Availability

All data generated during the current study are available from the corresponding authors upon reasonable request.

## Supplementary Materials

Supplementary Materials Supplementary 1. Figure S1: collagen I gel, ihAEpiC, and HUVEC morphologies in the microfluidic chip. For collagen I, it could be perfused into the central channel with liquid status and form a stable and uniform structure with a gel status after solidification. For ihAEpiC and HUVEC, the pictures showed the cell morphologies after being seeded. They could maintain these cell morphologies and grow into confluence after three-day culture. Figure S2: permeability measurement of 10 *μ*M 4 kDa FITC-dextran was taken with an average fluorescent intensity of different sites of the microchip. The distance of the detection site from the pillars is around 0.5 mm. The data means the average fluorescent intensity of the A, B, C, and D area varying with time. Fluorescent intensity of area A kept relatively stable because this site was the start position of the excess FITC-dextran. Fluorescent intensity of area B reached a platform at 30 min, which meant that FITC-dextran could permeate from site A to site B within 30 min. At 60 min, FITC-dextran finished the permeation process from site B to site C. For site D, it took about 120 min to finish the permeation process from site C to site D. That is to say, the collagen I gel could let macromolecules through based on its porous structure. This laid a fundament for biochemical communications between two kinds of cell cultured in two cell compartments separated by collagen gel. Figure S3: cells were stained with calcein AM (live cells, shown in green) and PI (dead cells, shown in red) for two modes: static cell culture mode and dynamic cell culture mode. Figure S4: dynamic flow cell culture was achieved with a CO_2_ incubator equipped with a flow injection pump. Tubes could be inserted into the incubator to connect chips through a customized hole on the back. Figure S5: permeability measurement of 10 *μ*M 4 kDa FITC-dextran was taken with an average fluorescent intensity of different sites of the microchip. For this microchip, ihAEpiC and HUVEC were cocultured for three days and formed an alveolar-capillary barrier together with collagen gel. The data means the average fluorescent intensity of the A, B, C, and D area varying with time. Fluorescent intensity of area A kept relatively stable because this site was the start position of the excess FITC-dextran. Fluorescent intensity of area B increased in the first 180 min. Compared with diffusion time of 30 min for bare microchip without cells (Figure S2), this proved a barrier function of ihAEpiC for the macromolecule permeability. For site C and site D, the average fluorescent intensity increased continuously in 720 min, a time point for medium refreshment, which meant that HUVEC also had a barrier function (compared with Figure S2). Figure S6: alveolar epithelial cells stimulated with air were stained with FM1-43 dye (yellow). After that, cell nuclei were stained with Hoechst dye (blue) as a position reference. Figure S7: alveolar epithelial cells treated with air stimulation were stained with FM1-43 dye after 12 h. Figure S8: endothelial cell morphology was recorded under an inverted microscope with a medium flow rate of 60 *μ*L/h. Cell elongation and alignment tendency were observed after 120 min with continuous medium flow. Figure S9: actin was immunofluorescently stained with TRITC-phalloidin for endothelial cells with continuous medium flow after 4 h and 12 h. Figure S10: actin was immunofluorescently stained with TRITC-phalloidin for epithelial cells with continuous medium flow for different periods of time at a flow rate of 60 *μ*L/h. Figure S11: the established alveolus-on-a-chip was cultured under an inverted microscope with a live cell culture and imaging station. The cyclic air simulation was achieved by using a pressure pump. The flow mechanical stress was achieved by using an injection pump with a water bath for medium warm-up. Figure S12: structural formula of poly(I:C). Figure S13: quantitative fluorescence intensity of the GFP expressed in the host cells for the viral infection. (a) The average fluorescence intensity of GFP expressed in ihAEpiC and HUVEC with different viral concentrations in cell medium; (b) the average fluorescence intensity of GFP expressed in HUVEC for the viral infection with different antibody concentrations. Figure S14: the size data of the microchip. Figure S15: photos of the alveolus-on-a-chip: (A) and (B) show the actual size of the chip with about 25 mm × 30 mm × 10 mm; (C) cell seed and culture in the microfluidic chip; (D) introduction of mechanical force strain. Scheme S1: workflow diagram of the microfluidic chip fabrication process. Supplementary 2. Video S1: static cell culture mode: cells were stained with calcein AM. Yellow arrows indicate a live cell division process. Images were captured with an inverted microscope with 1 pic/30 min. Supplementary 3. Video S2: dynamic cell culture mode: cells were stained with calcein AM. Yellow arrows indicate a live cell division process. Images were captured with an inverted microscope with 1 pic/30 min. Supplementary 4. Video S3: a sinusoidal cyclic air mechanical strain provided by a pressure pump was applied into ihAEpiC in the epithelial cell compartment. The produced cyclic pressure oscillation could be seen from periodic vibration of the collagen gel.
