# Supplementary material for: Biomimetic Alveolus-on-a-Chip for SARS-CoV-2 Infection Recapitulation
Source: Research (Wash D C). 2022 Feb 4;2022:9819154. doi: 10.34133/2022/9819154 (PMC8841031; doi:10.34133/2022/9819154)
Supplement: Supplementary Materials — Supplementary 1. Figure S1: collagen I gel, ihAEpiC, and HUVEC morphologies in the microfluidic chip. For collagen I, it could be perfused into the central channel with liquid status and form a stable and uniform structure with a gel status after solidification. For ihAEpiC and HUVEC, the pictures showed the cell morphologies after being seeded. They could maintain these cell morphologies and grow into confluence after three-day culture. Figure S2: permeability measurement of 10 μM 4 kDa FITC-dextran was taken with an average fluorescent intensity of different sites of the microchip. The distance of the detection site from the pillars is around 0.5 mm. The data means the average fluorescent intensity of the A, B, C, and D area varying with time. Fluorescent intensity of area A kept relatively stable because this site was the start position of the excess FITC-dextran. Fluorescent intensity of area B reached a platform at 30 min, which meant that FITC-dextran could permeate from site A to site B within 30 min. At 60 min, FITC-dextran finished the permeation process from site B to site C. For site D, it took about 120 min to finish the permeation process from site C to site D. That is to say, the collagen I gel could let macromolecules through based on its porous structure. This laid a fundament for biochemical communications between two kinds of cell cultured in two cell compartments separated by collagen gel. Figure S3: cells were stained with calcein AM (live cells, shown in green) and PI (dead cells, shown in red) for two modes: static cell culture mode and dynamic cell culture mode. Figure S4: dynamic flow cell culture was achieved with a CO2 incubator equipped with a flow injection pump. Tubes could be inserted into the incubator to connect chips through a customized hole on the back. Figure S5: permeability measurement of 10 μM 4 kDa FITC-dextran was taken with an average fluorescent intensity of different sites of the microchip. For this microchip, ihAEpiC and [file 9819154.f1.zip › Cao_Supplementary Information.docx]

Supplementary Information

Biomimetic alveolus-on-a-chip for SARS-CoV-2 infection recapitulation

**Ting Cao^1,2,3^, Changmin Shao^1,3^, Xiaoyu Yu^4^, Ruipei Xie^2^, Chen Yang^4^, Yulong Sun^1,2,3^, Shaohua Yang^4^, Wangjian He^4^, Ye Xu^4^, Qihui Fan^2^*, Fangfu Ye^1,2,3^***

^1^Oujiang Laboratory (Zhejiang Lab for Regenerative Medicine, Vision and Brain Health), Wenzhou, Zhejiang 325001, China.

^2^Beijing National Laboratory for Condensed Matter Physics, Institute of Physics, Chinese Academy of Sciences, Beijing 100190, China.

^3^Wenzhou Institute, University of Chinese Academy of Sciences, Wenzhou, Zhejiang 325001, China.

^4^School of Mechanical Engineering & Automation, Beihang University, Beijing 100191, China.

Correspondence should be addressed to Qihui Fan; fanqh@iphy.ac.cn and Fangfu Ye; fye@iphy.ac.cn.


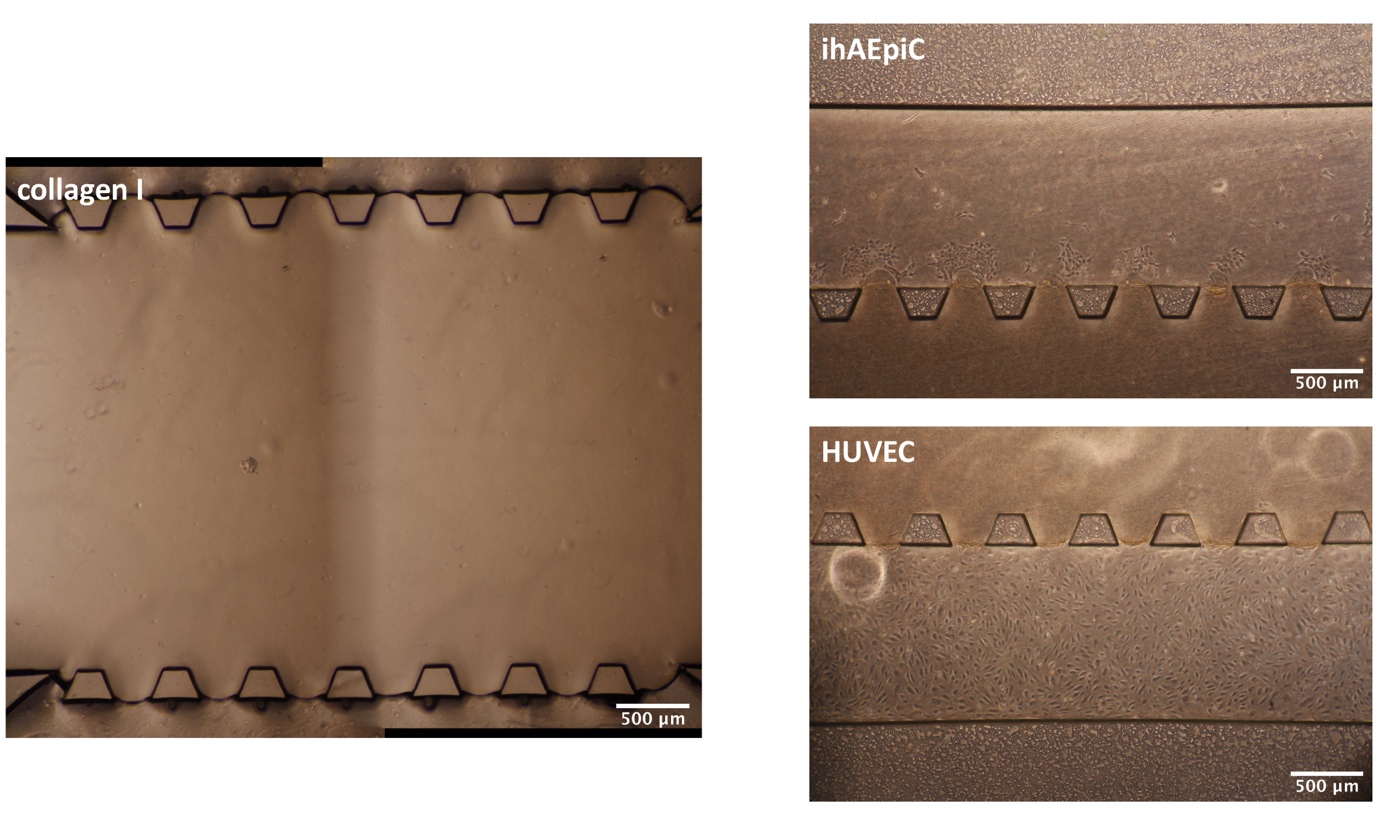


Figure S1. Collagen I gel, ihAEpiC and HUVEC morphologies in the microfluidic chip. For Collagen I, it could be perfused into the central channel with liquid status and form a stable and uniform structure with a gel status after solidification. For ihAEpiC and HUVEC, the pictures showed the cell morphologies after seeded. They could maintain these cell morphologies and grow into confluence after three days culture.


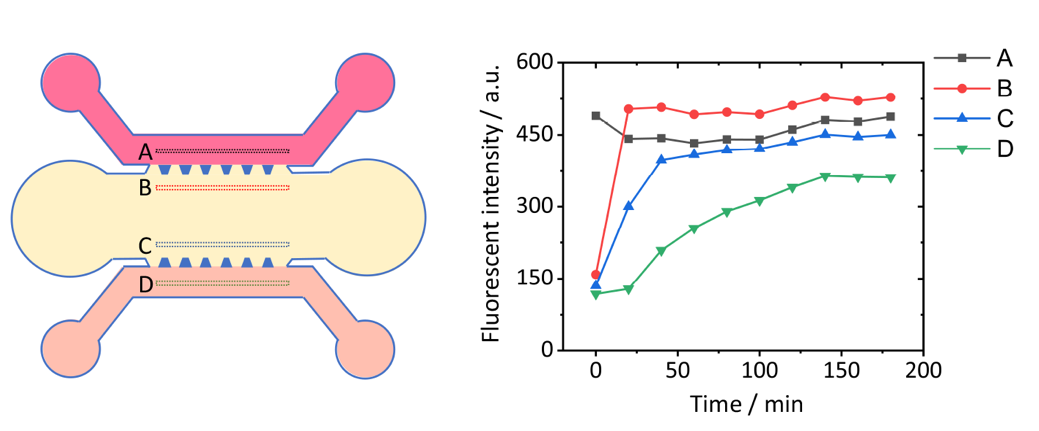


Figure S2. Permeability measurement of 10 µM 4 kDa FITC-dextran was taken with an average fluorescent intensity of different sites of the microchip. The distance of the detection site from the pillars is around 0.5 mm. The data means the average fluorescent intensity of the A, B, C and D area varying with time. Fluorescent intensity of area A kept relatively stable because this site was the start position of the excess FITC-dextran. Fluorescent intensity of area B reached a platform at 30 min, which meant FITC-dextran could permeate from site A to site B within 20 min. At 40 min, FITC-dextran finished the permeation process form site B to site C. For site D, it took about 140 min to finish the permeation process form site C to site D. That is to say, the Collagen I gel could let macromolecules through based on its porous structure. This laid a fundament for biochemical communications between two kinds of cell cultured in two cell compartments separated by collagen gel.


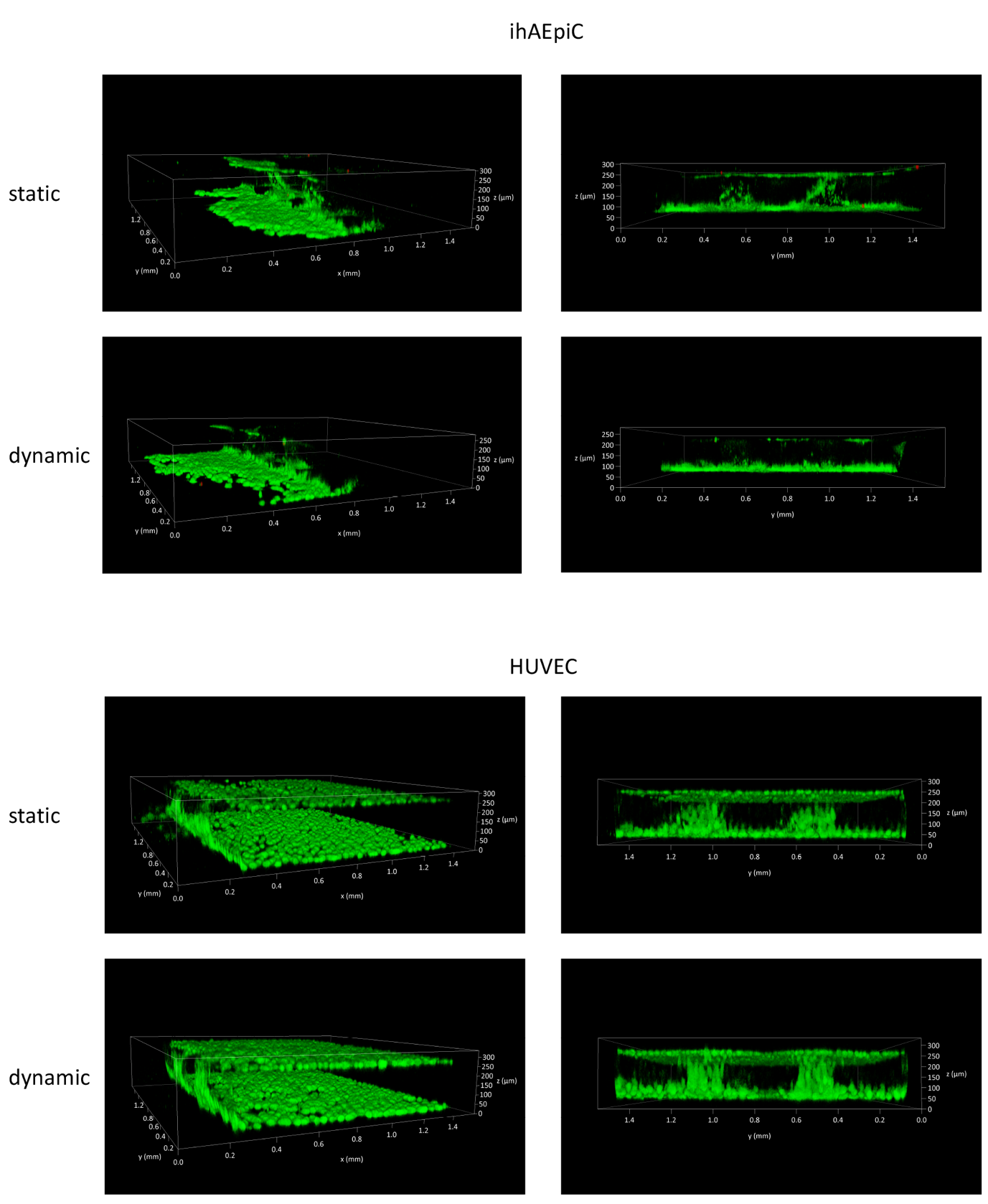


Figure S3. Cells were stained by Calcein AM (live cells, shown in greed) and PI (dead cells, shown in red) for two modes: static cell-culture-mode and dynamic cell-culture-mode.


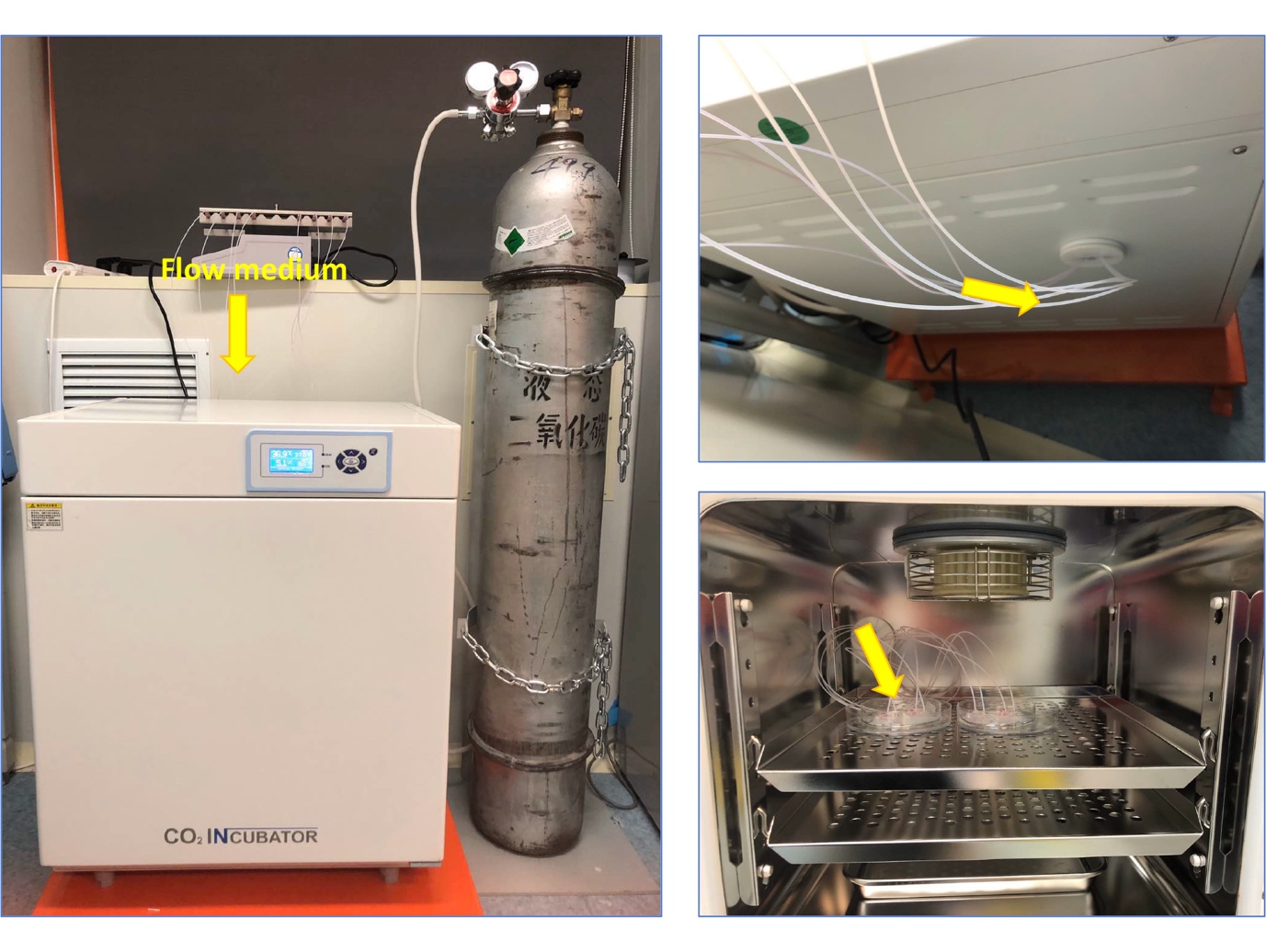


Figure S4. Dynamic flow cell culture was achieved with a CO2 incubator equipped with a flow injection pump. Tubes could be inserted into the incubator to connect chips through a customized hole on the back.


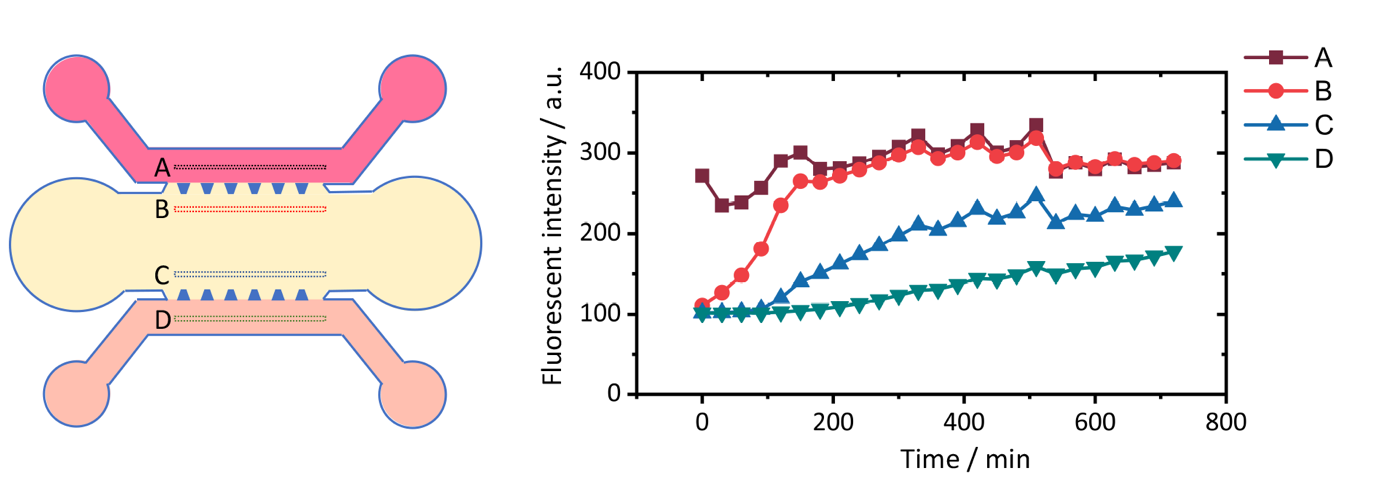


Figure S5. Permeability measurement of 10 µM 4 kDa FITC-dextran was taken with an average fluorescent intensity of different sites of the microchip. For this microchip, ihAEpiC and HUVEC were cocultured for three days and formed an alveolar-capillary barrier together with collagen gel. The data means the average fluorescent intensity of the A, B, C and D area varying with time. Fluorescent intensity of area A kept relatively stable because this site was the start position of the excess FITC-dextran. Fluorescent intensity of area B increased in first 180 min. Compared with diffusion time of 30 min for bare microchip without cells (Figure S2), this proved a barrier function of ihAEpiC for the macromolecule permeability. For site C and site D, the average fluorescent intensity increased continuously in 720 min, a time point for medium refreshment, which meant HUVEC also had a barrier function (compared with Figure S2).


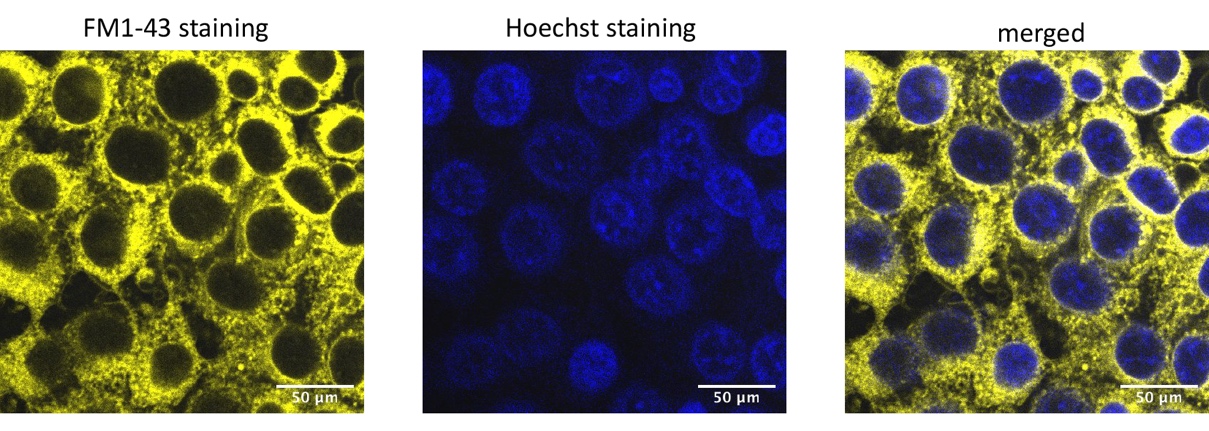


Figure S6. Alveolar epithelial cells stimulated with air were stained by FM1-43 dye (yellow). After that, cell nucleuses were stained by Hoechst dye (blue) as a position reference.


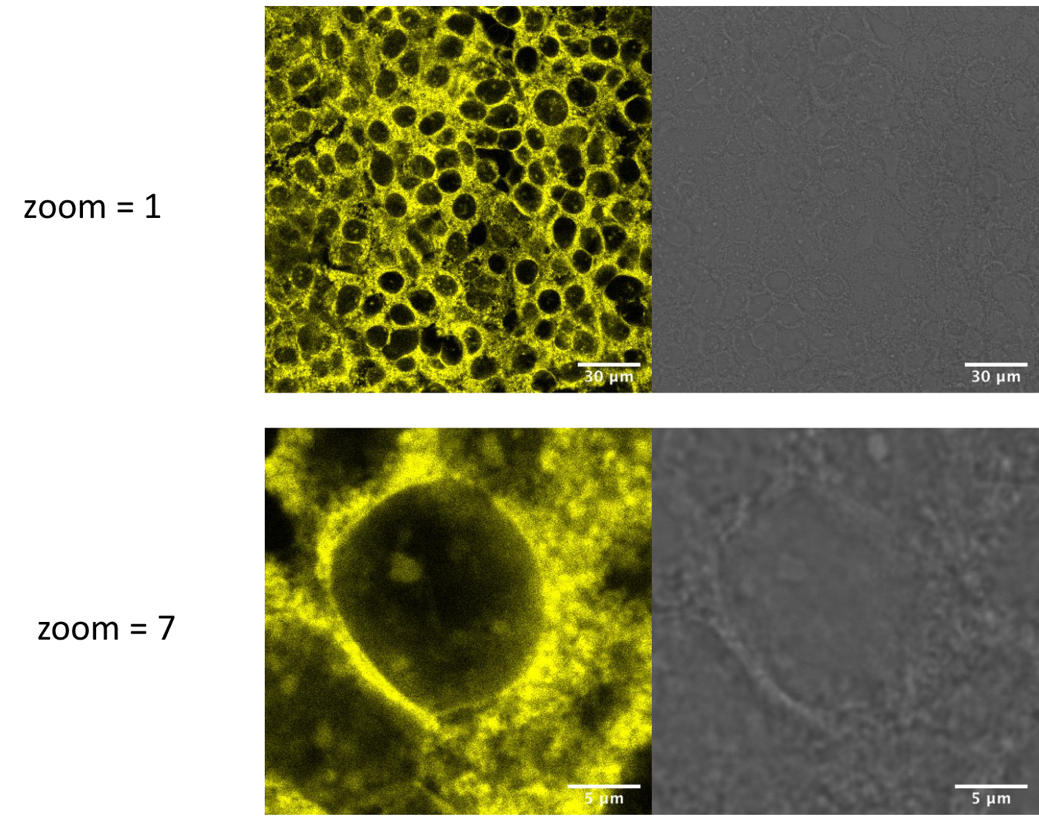


Figure S7. Alveolar epithelial cells treated with air stimulation were stained by FM1-43 dye after 12 h.


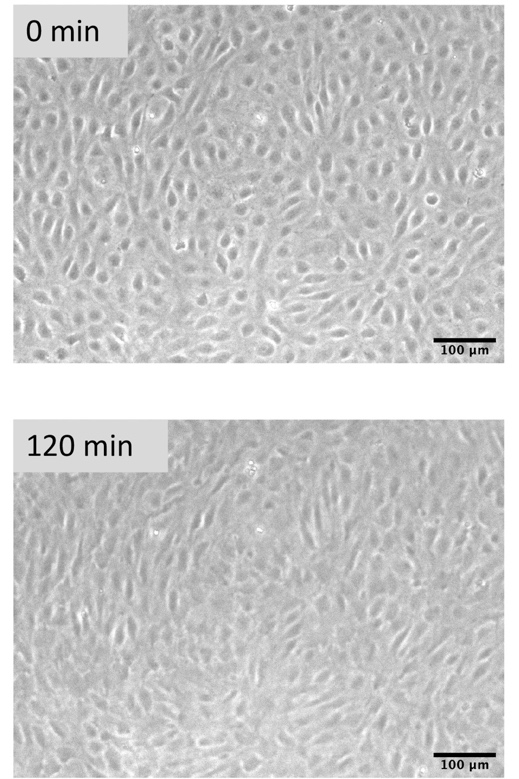


Figure S8. Endothelial cell morphology was recorded under an inverted microscope with a medium flow rate of 60 µL/h. Cell elongation and alignment tendency were observed after 120 min with continuous medium flow.


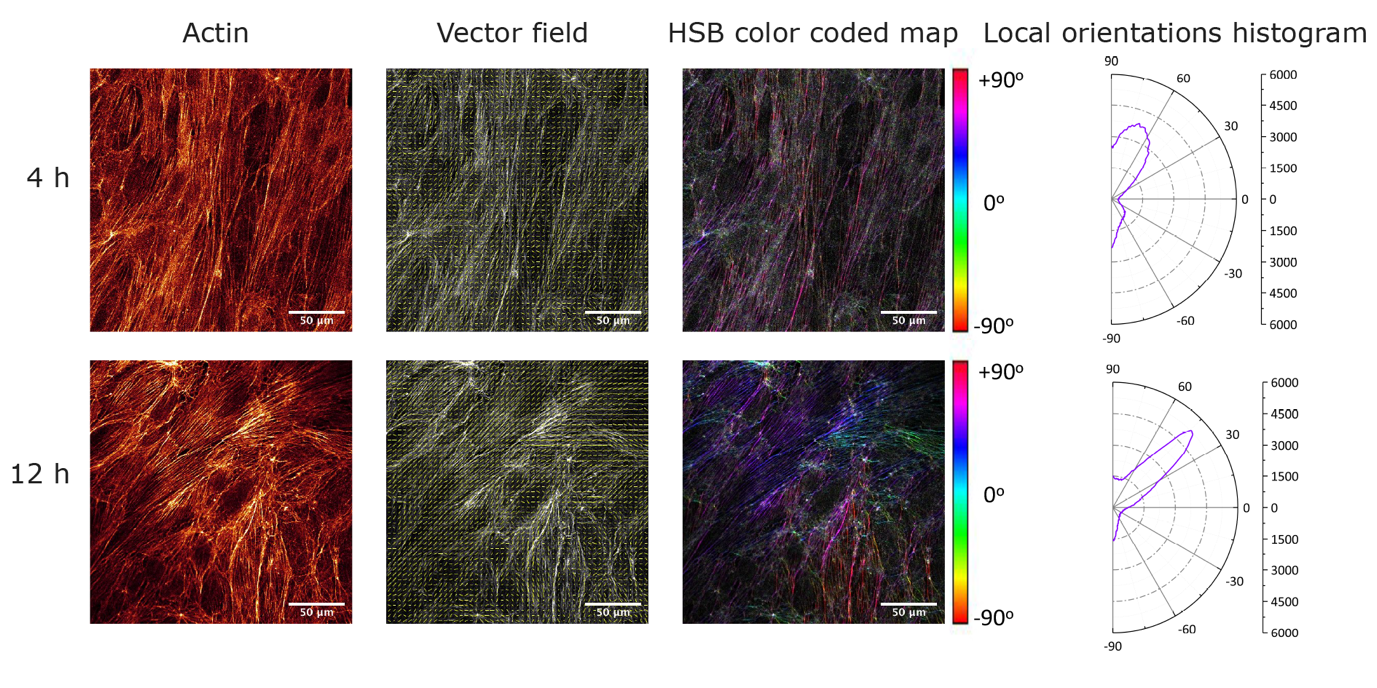


Figure S9. Actin was immunofluorescent stained by TRITC-Phalloidin for endothelial cells with continuous medium flow after 4 h and 12 h.


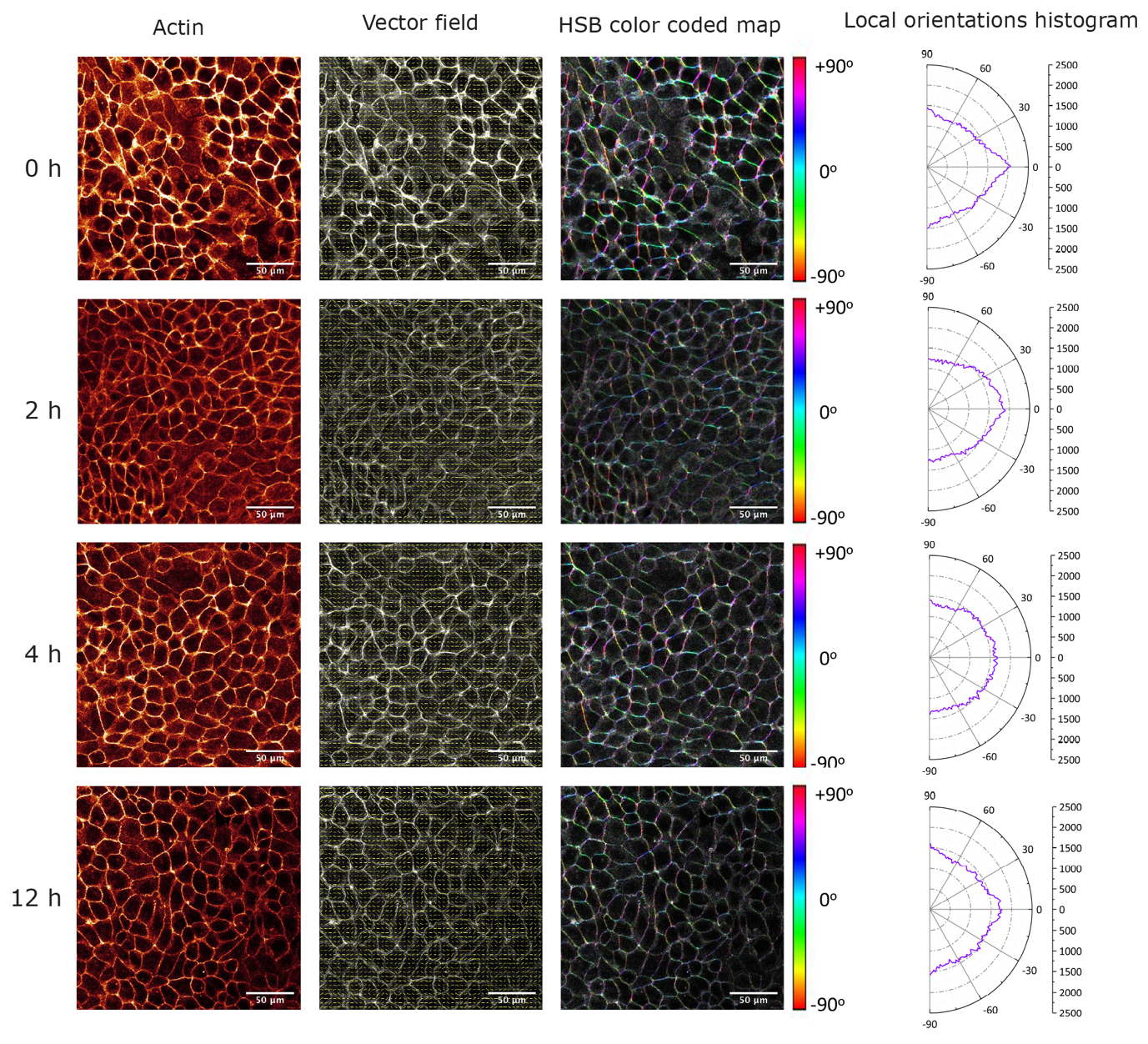


Figure S10. Actin was immunofluorescent stained by TRITC-Phalloidin for epithelial cells with continuous medium flow for different period of time at a flow rate of 60 µL/h.


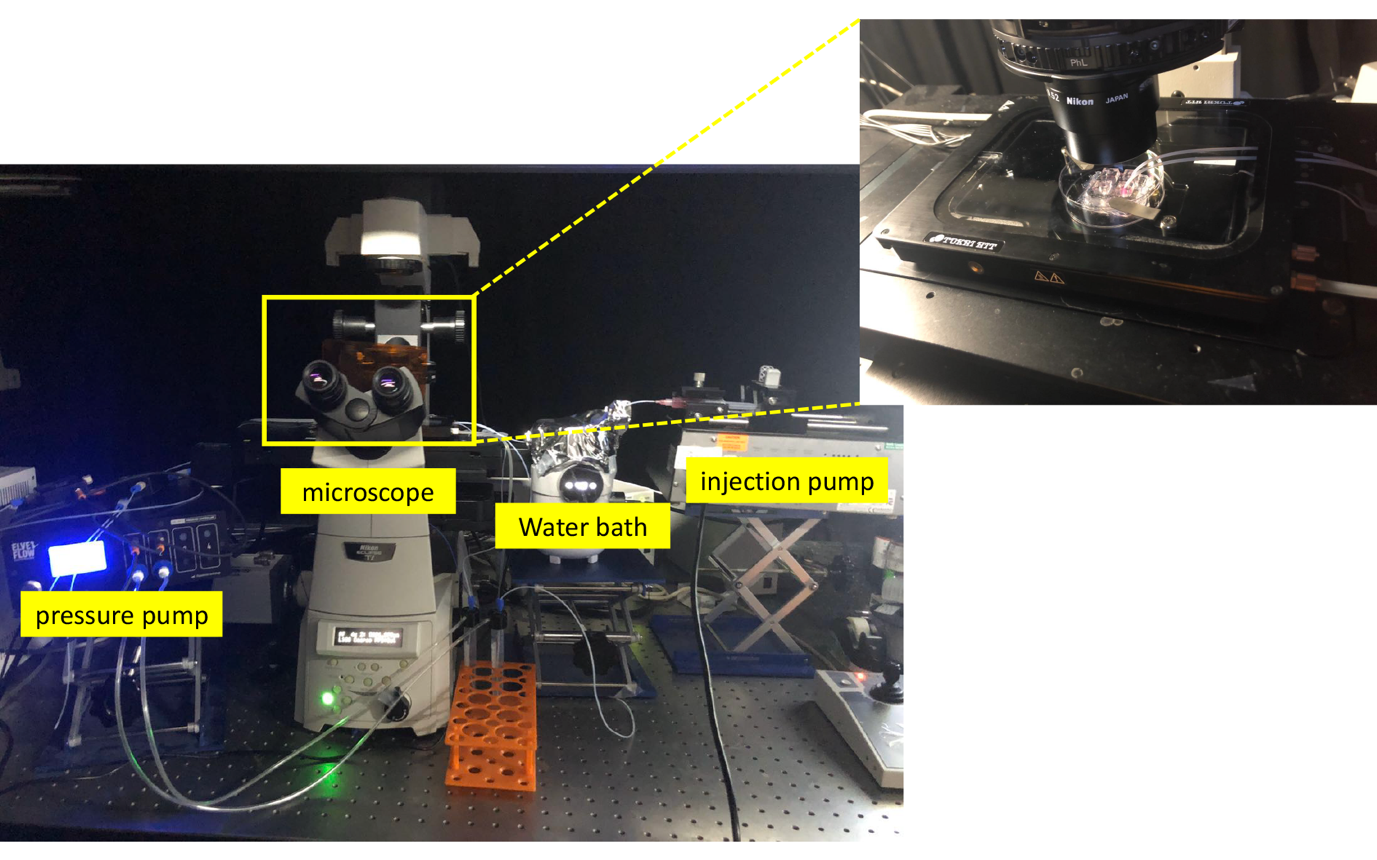


Figure S11. The established alveolus-on-a-chip was cultured under an inverted microscope with a live cell culture and imaging station. The cyclic air simulation was achieved by a pressure pump. The flow mechanical stress was achieved by a injection pump with a water bath for medium warm-up.


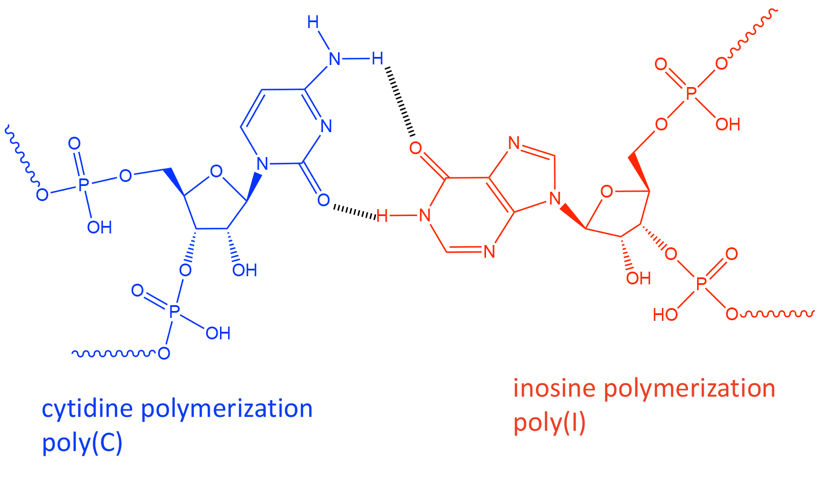


Figure S12. Structural formula of poly(I:C).


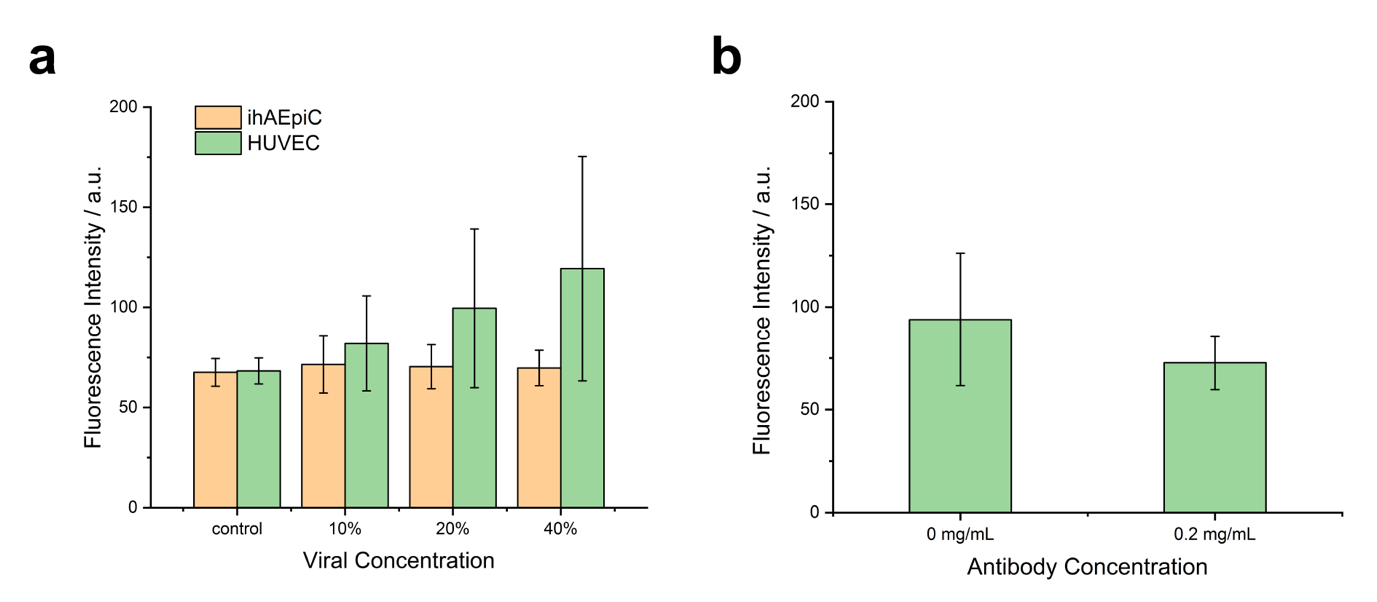


Figure S13. Quantitative fluorescence intensity of the GFP expressed in the host cells for the viral infection. (a) The average fluorescence intensity of GFP expressed in ihAEpiC and HUVEC with different viral concentrations in cell medium; (b) The average fluorescence intensity of GFP expressed in HUVEC for the viral infection with different antibody concentrations.


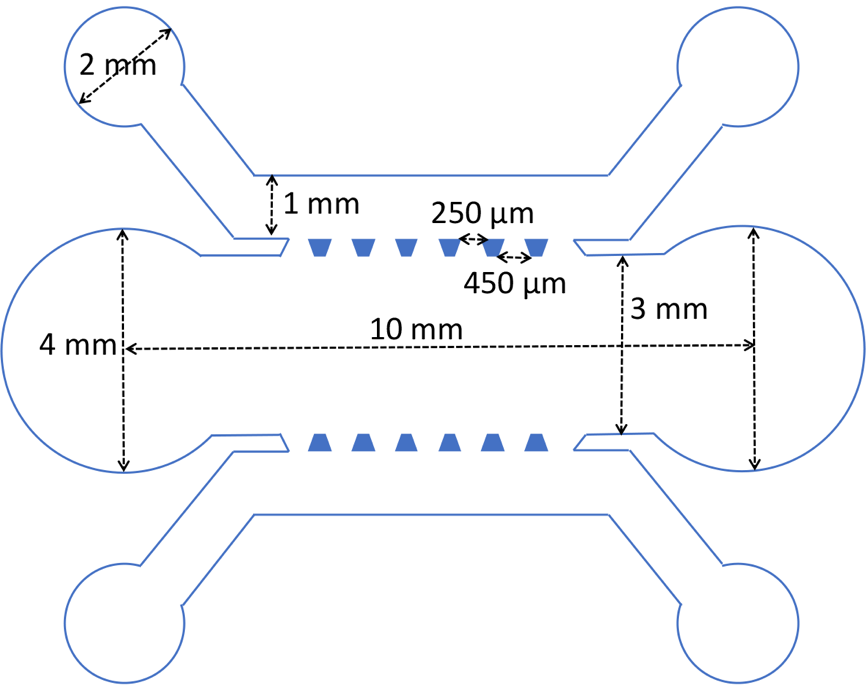


Figure S14. The size data of the microchip.


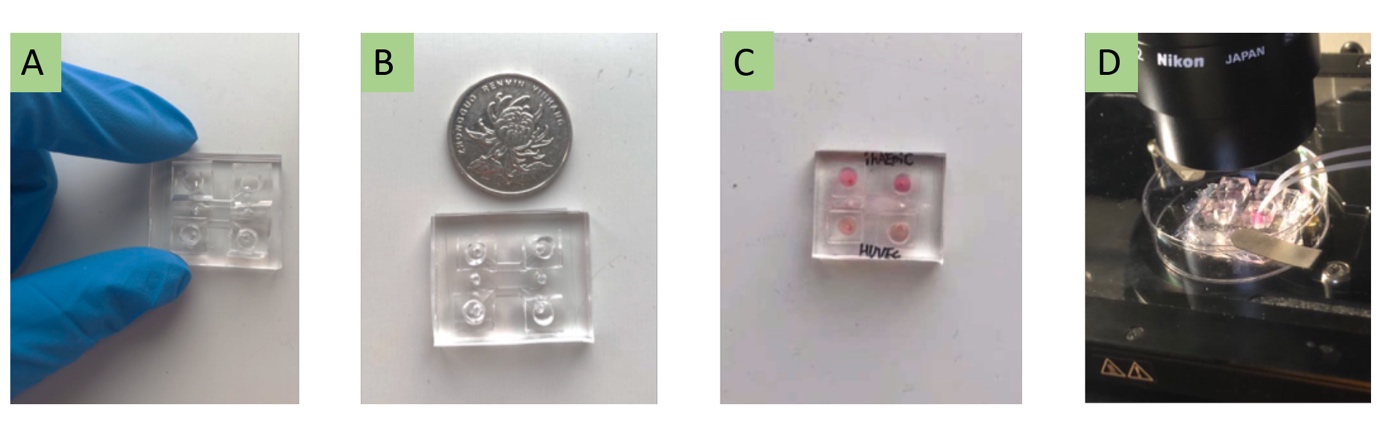


Figure S15. Photos of the alveolus-on-a-chip: (A) and (B) show the actual size of the chip with about 25mm x 30 mm x 10 mm; (C) Cell seed and culture in the microfluidic chip; (D) Introduce of mechanical force strain.


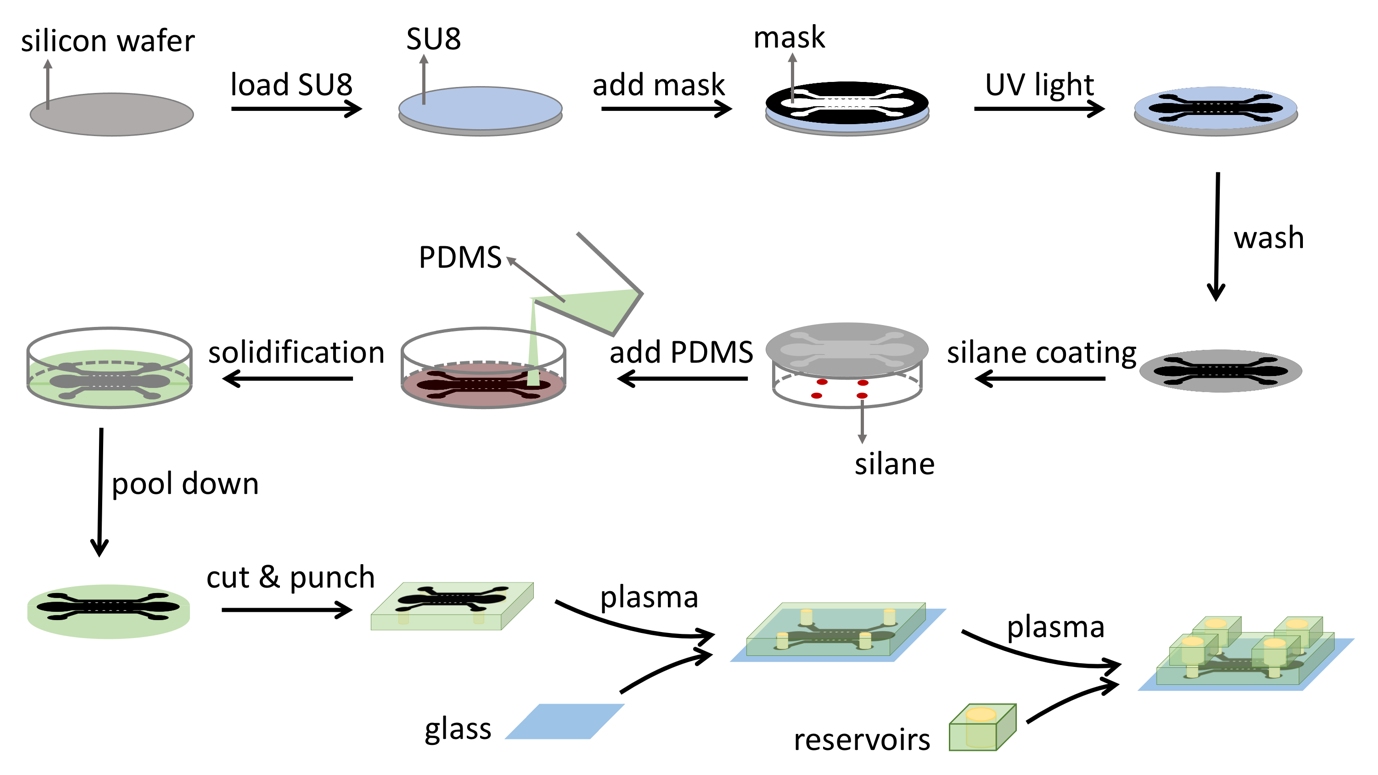


Scheme S1. Workflow diagram of the microfluidic chip fabrication process.
